# Use of Anæsthetic Agents

**Published:** 1848-10

**Authors:** 


*Use of Anæsthetic Agents.*—Quite a revulsion of opinion seems to have taken place in England in regard to the use of anæsthetic agents. The editor of the *London Medical Gazette*, in an editorial article (No. for July 16th, 1848,) commenting on the case of Mr. Badger, (noticed in our last number, p. 84,) remarks:—"The deceased was a healthy, muscular young man, who, according to the testimony of his father, had suffered from no difficulty of breathing, or any other apparent disease. The inspection of the body, however, revealed a diseased state of the heart and liver, although not sufficient to account for sudden death. Hence we arrive at the conclusion that a young and healthy-looking person, whose appearance and previous habits of life would create no suspicion of the existence of latent organic disease, may still be in such a condition of body that the respiration of the vapor will operate upon him like a fatal poison. It is not here as with a liquid or solid taken into the stomach—the poison enters at once into the circulation, and penetrates through the whole of the system; and but a few minutes elapse between apparently perfect health and the death of the patient. The circumstances under which the poison is administered do not, in these unfavorable cases, admit of the application of any remedy. The attempt to abstract blood has uniformally failed. Art is powerless in dealing with the poisonous effects of this vapor. It may be said, and we doubt not the truth of the statement, that hundreds, nay, thousands of persons, young and healthy-looking like the deceased, have inhaled this vapor without any such disastrous effects following. We have, however, heard of some very narrow escapes, even where precaution and skill of the best kind had been employed in its administration; and doubtless the experience of many of our readers will furnish them with cases corroborative of this remark. But the death of only one person in a thousand, when the vapor has been skillfully administered—and there was nothing in the patient's aspect or account of himself to induce the operator to withhold his consent to its employment—becomes a most serious matter. There should be some extraordinary advantage or benefit to the individual to justify such a fearful risk; but the advantage, if any, in reference to the dental art, is the alleviation of pain merely for a few minutes; and the naked question now to be considered is, will any operator feel himself justified, after the case of Mr. Badger, in employing this dangerous vapor for the annulling of pain in the extraction of teeth? If latent disease of the heart or liver could always be clearly diagnosed in a patient, we should not be called upon to put this question: but as Mr. Badger's case proves that a most experienced man like Mr. Robinson saw nothing about the deceased to justify his refusal to employ chloroform, it is clear that the most skillful dentist may be working in the dark, and thus unconsciously be the means of sacrificing life for the sake of humoring a patient by annulling a degree of pain which every healthy adult should be able to bear. The facts of this case have, however, a bearing far beyond dentistry. We consider that our remarks apply to all the minor operations of surgery."

☞ The editor of the *Medical Times*, in an editorial article, (Number for 22d July, 1646,) uses still more decided language in regard to the use of anæsthetic agents.

"The fate of chloroform," he asserts, "is sealed, at least so far as relates to its employment in minor surgical operations. The deaths which have occurred to persons under its influence, both in England, France, and America, have incontestably proved that it is a medicine which cannot be administered without great risk. Anæsthetic agents may be fairly said to have had their day, and henceforth they will cease to be employed, either in surgery or midwifery, except under peculiar circumstances, and by persons of more than ordinary courage. The law, up to the present time, has not interposed to stop the perilling of human life by the inhalation of ether and chloroform: but another death or two will rouse it into action, and surgeons, under whose sanction these agents may hereafter be administered, will be exposed to the danger of a prosecution for manslaughter.

"The great experiment has now been fairly tried, whether patients about to submit to surgical operations can be rendered insensible to pain by certain agents, and their lives not perilled. For a time all seemed to promise well, and the first death which occurred, was attributed to some other cause than the administration of chloroform or ether. The scientific world was unwilling to admit that agents apparently so valuable, should possess attributes which would prevent their general employment. Thus brandy, rather than chloroform, was stigmatized with the guilt of Hannah Greener's death: and the druggist's apprentice, who expired after inhaling the anæsthetic agent, merely to produce a momentary pleasure, was said to have given up the ghost for the want of breath, his face being enveloped in the napkin from which he inhaled the much-loved gas. Facts, however, have since accumulated, which forbid us to doubt that, under certain circumstances, death will follow the exhibition of chloroform and ether.

"Could these circumstances be clearly ascertained beforehand by the surgeon, all difficulty would vanish, and science would be justly proud of the discoveries of Morton and of Simpson. Pain, however, seems as yet to be indissolubly linked to human nature, and science has labored in vain to produce its extinction. Chloroform and ether, which appeared for a season to be two of the most important gifts ever given to man, have proved, to some, fatal poisons—penetrating in an instant the whole system —attacking the vital energies, and compelling them to yield to death, though apparently uninjured previously by disease.

The way in which anæsthetic agents are introduced into the system, renders the resources of our art powerless in any attempts to counteract their poisonous influence. In no single instance that we know of, has the effort to abstract blood been successful; nor are we disposed to imagine that much good would result from bleeding, if the venous and arterial currents were not completely stagnated. Blood appears to lose those characteristics which are essential to life, assuming a dark color in the arteries, while the fluid in the veins becomes mixed with air. This latter circumstance has been noticed in nearly every fatal case, and is a phenomenon which as yet has not been satisfactorily accounted for. In the case of the French female, M. Gorré, the surgeon who administered the chloroform, endeavored to account for the presence of air in the veins, from the possibility of its being introduced at the time of performing the operation, which was only that of opening an abscess for the extraction of a foreign body. Like a zealous friend of anæsthetic agents he suggests the idea that the patient died from this cause, and not from the inhalation of chloroform. M. Velpeau, with great propriety, controverts this opinion, as death appears to have actually taken place before the operation was commenced; yet he advances an opinion equally as improbable as that of M. Gorré, viz., that, as the autopsy was not performed till twenty-four hours after death, putrefaction might have been the cause of the air in the veins.

"In the American case, though the body was not examined till twenty-four hours after death, putrefaction could not have been the means of generating the gasses found in the sinuses of the dura matter, as in the month of February, in Cincinnati, the temperature of the air is not sufficiently high to produce decomposition sufficiently rapid to generate gasses in the veins even of a person who died in the midst of health.

"Looking over the cases, we are led to conclude that the quantity of chloroform exhibited has very little to do with the fatal results. In Mr. Robinson's patient, a drachm and a half only was placed in the apparatus; in the American case, there was probably not more administered; while the patient of M. Gorre had only placed upon the handkerchief fifteen or twenty drops.

"In all the fatal cases, death occurred without any previous signs of disease. Had the patients presented anything unusual in their appearance, it is likely that the surgeons would have detected it; and it is this circumstance that renders the administration of chloroform so dangerous.

"Hence it becomes the members of the medical profession to use with the greatest circumspection, anæsthetic agents. In the minor operations of surgery they are now inadmissable, and even in the great operations, it becomes an important question whether the risk incurred will justify their employment.

"Since writing the above remarks, on looking over the *Gazette Medicale* of the 15th of July, we find a case recorded of a corpulent young man, twenty-four years of age, admitted into the Hôpital Beaujon, on the 25th of June, and who suffered amputation of the thigh, in consequence of a gun-shot wound. The patient inhaled the chloroform, and before the operation was concluded, sensibility appeared to be returning, when M. Robert, the surgeon, ordered some more chloroform to be inhaled; within a quarter of a minute the respiration became stertorous, the face and lips pale, the pupils dilated, and the head fell upon the shoulder. Powerful friction, and irritating the pituitary membrane seemed for a moment, in this case, to produce signs of returning sensibility. She patient, however, soon expired.

"Here, again, the surgeon was willing to suppose that the anæsthetic agent was not the cause of death, but rather the peculiar condition of the patient from the gun-shot wound which he had received. Be this as it may, the fact is now established that anæsthetic agents cannot be employed without, in many cases, endangering the lives of those who are placed under their influence."—*Medical News.*

